# Increased serum soluble Fas after major trauma is associated with delayed neutrophil apoptosis and development of sepsis

**DOI:** 10.1186/cc9965

**Published:** 2011-01-13

**Authors:** Adnana Paunel-Görgülü, Sascha Flohé, Martin Scholz, Joachim Windolf, Tim Lögters

**Affiliations:** 1 Department of Trauma and Hand Surgery, University Hospital Düsseldorf, Moorenstrasse 5, D-40225 Düsseldorf, Germany

## Abstract

**Introduction:**

Deregulated apoptosis and overshooting neutrophil functions contribute to immune and organ dysfunction in sepsis and multiple organ failure (MOF). In the present study, we determined the role of soluble Fas (sFas) in the regulation of posttraumatic neutrophil extrinsic apoptosis and the development of sepsis.

**Methods:**

Forty-seven major trauma patients, 18 with and 29 without sepsis development during the first 10 days after trauma, were enrolled in this prospective study. Seventeen healthy volunteers served as controls. Blood samples from severely injured patients were analyzed at day 1, day 5 and day 9 after major trauma. sFas levels, plasma levels of neutrophil elastase (PMNE) and levels of interleukin (IL)-6 were quantified by enzyme-linked immunosorbent assay and related to patients' Sequential Organ Failure Assessment (SOFA) score and Multiple Organ Dysfunction Score (MODS). Neutrophil apoptosis was determined by propidium iodide staining of fragmented DNA and flow cytometry. sFas-mediated effects on neutrophil apoptosis were investigated in cells cultured with agonistic anti-Fas antibodies in the presence of recombinant sFas, sFas-depleted serum or untreated serum from septic patients.

**Results:**

Serum levels of sFas in patients who later developed sepsis were significantly increased at day 5 (*P *< 0.01) and day 9 (*P *< 0.05) after trauma compared with patients with uneventful recovery. Apoptosis of patient neutrophils was significantly decreased during the observation period compared with control cells. Moreover, Fas-mediated apoptosis of control neutrophils was efficiently inhibited by recombinant sFas and serum from septic patients. Depletion of sFas from septic patient sera diminished the antiapoptotic effects. In septic patients, sFas levels were positively correlated with SOFA at day 1 (*r *= 0.7, *P *< 0.001), day 5 (*r *= 0.62, *P *< 0.01) and day 9 (*r *= 0.58, *P *< 0.01) and with PMNE and leukocyte counts (*r *= 0.49, *P *< 0.05 for both) as well as MODS at day 5 (*r *= 0.56, *P *< 0.01) after trauma.

**Conclusions:**

Increased sFas in patients with sepsis development impairs neutrophil extrinsic apoptosis and shows a positive correlation with the organ dysfunction scores and PMNE. Therefore, sFas might be a therapeutic target to prevent posttrauma hyperinflammation and sepsis.

## Introduction

Major trauma is frequently associated with activation of polymorphonuclear neutrophils and systemic inflammation. Normally, the life span of neutrophils, which constitute an important line of innate host defense, is limited by apoptosis [1]. During inflammation, neutrophils rapidly migrate from the blood into solid tissues to protect organs from invading bacteria [2]. However, the life span of these neutrophils is prolonged, resulting in lung [3], liver [4] and kidney [5] injury. Further, neutrophil accumulation in the lung and distant organs represents a characteristic finding in patients dying of sepsis [6]. Neutrophils may cause tissue damage by the secretion of reactive oxygen species (ROS) and proteolytic enzymes, of which neutrophil elastase (PMNE) is the most abundant [7,8]. There is strong evidence for a direct correlation between impaired neutrophil apoptosis and overshooting inflammation [9].

Apoptosis is tightly regulated and might be activated via membrane-bound "death" receptors, such as Fas (extrinsic pathway), or via the mitochondrion (intrinsic pathway). Fas/Fas ligand (FasL) signaling has emerged as an important cellular pathway regulating the induction of apoptosis in a wide variety of tissues as well as activated immune cells [10,11], thus playing a crucial role in the resolution of inflammatory responses [9]. The Fas receptor, also designated as CD95 or Apo-1, is a type I cell surface glycoprotein which belongs to the tumor necrosis factor (TNF) receptor superfamily of membrane receptors and has a broad distribution on various tissues [12]. The Fas molecule could occur as a cell surface receptor as well as a soluble protein. The soluble form of Fas (sFas) is derived either by alternative splicing from the membrane form or by proteolytic cleavage of membrane-bound receptors [13,14]. sFas seems to play an important role as a signaling molecule. It has been suggested that sFas modifies ligand concentration, downregulates membrane receptor numbers and specifically inhibits ligand-receptor association in the extracellular space, thus preventing the induction of apoptosis in Fas-bearing target cells. Furthermore, expression of sFas in mice leads to an autoimmune syndrome, and elevated levels of sFas have been found in some patients with autoimmune diseases [13]. FasL is a type II integral membrane protein which is more restricted and tightly regulated in its expression [12], and the procession by a matrix metalloproteinase results in protein cleavage and release of the extracellular domain [15]. The biologically active soluble form of FasL (sFasL) as well as agonistic anti-Fas antibodies are capable of inducing cytotoxicity, hepatocyte destruction and mortality in mice through the interaction with hepatocyte Fas [16,17] and might contribute to systemic tissue destruction during inflammation [18].

Neutrophils express both Fas and its endogenous ligand FasL on their surface, and therefore Fas-FasL interaction may represent a mechanism of autocrine/paracrine neutrophil death regulation [19]. Several previous studies have reported reduced Fas-mediated apoptosis in neutrophils obtained from humans with systemic inflammatory response syndrome (SIRS), burn injuries or surgical trauma [20,21], without elucidating the regulatory mechanisms of the disturbed apoptosis.

In the current study, we provide evidence for serum sFas-mediated inhibition of neutrophil apoptosis and have determined the prognostic value of sFas in posttraumatic sepsis.

## Materials and methods

### Patients

Forty-seven patients were enrolled in this prospective study. Study approval was obtained from the Ethics Review Board of the University of Düsseldorf (Düsseldorf, Germany). Patients with blunt or penetrating multiple injuries who were admitted to our Level I Trauma Center with an Injury Severity Score (ISS) >16, intensive care unit (ICU) stay >3 days and ages 18 years and older were enrolled in this study. Written, informed consent was obtained from all participants or their legal representatives if the patients were unconscious. Exclusion criteria were death of the patient on the day of admission or within the first 2 days on the ICU and withdrawal of patient consent. In addition, patients with known preexisting immunological disorders or systemic immunosuppressive medication were excluded. The severity of injury was assessed by using the ISS, which is based on the Abbreviated Injury Scale (AIS) [22], on admission to the emergency room. SIRS and sepsis were defined using the criteria outlined in 2005 by the International Sepsis Forum [23]. SIRS was considered to be present when patients' conditions fulfilled more than one SIRS criterion. Patients were determined as septic if they fulfilled criteria for SIRS and had a proven source of infection. To evaluate organ dysfunction and/or failure, the Sequential Organ Failure Assessment (SOFA) and Multiple Organ Dysfunction (MOD) scores [24] were determined. Severe sepsis referred to sepsis complicated by organ dysfunction. Organ dysfunction has been defined using the definition by the SOFA score with >2 points for at least one system (respiratory, coagulation, liver, cardiovascular, central nervous or renal system). Septic shock was defined as sepsis with acute persistent circulatory failure unexplained by other causes (>2 points in SOFA score for the cardiovascular system).

The patients included in this study did not receive low-dose hydrocortisone therapy as routine adjuvant treatment for septic shock. Seventeen healthy volunteers served as the control group.

Blood was collected from healthy volunteers and daily from patients from the day of admission until day 9. Heparinized blood was immediately used after collection for neutrophil isolation. In parallel, sera and plasma were harvested by centrifugation and stored at -80°C until further processing.

### Quantification of sFas, sFasL, IL-6 and PMNE by ELISA

sFas (detection limit <47 pg/mL), sFasL (detection limit <12 pg/mL) (both evaluated by Hoelzel Diagnostika, Cologne, Germany) and interleukin (IL)-6 (detection limit <0.70 pg/mL) (evaluated by R&D Systems, Wiesbaden-Nordenstadt, Germany) were measured in serum and PMNE (detection limit 3 ng/mL) (evaluated by Milenia Biotec, Gießen, Germany) in plasma samples by using commercially available enzyme-linked immunosorbent assay (ELISA) kits according to the manufacturer's instructions.

### Isolation of human neutrophils

Human neutrophils were isolated by discontinuous density gradient centrifugation using Percoll medium (Biochrom, Berlin, Germany) as previously described [25]. After hypotonic lysis to remove contaminating erythrocytes, cells were suspended in phosphate-buffered saline (PBS). Purity and viability were routinely >95% as assessed by forward and side scatter characteristics of FACScan (BD Biosciences, Heidelberg, Germany) and Trypan blue exclusion, respectively.

### Immunoprecipitation of sFas from patient serum

The monoclonal anti-Fas antibody clone ZB4 (2 μg; Millipore, Schwalbach, Germany) was mixed with 40 μL of Protein G Plus/Protein A-Agarose beads (Calbiochem, Darmstadt, Germany) and incubated for 3 hours with gentle shaking. Then pooled serum from four septic patients was added and incubated for an additional 17 hours at 4°C with gentle shaking. Bound immune complexes were spun down, and the supernatant was stored at -80°C until use.

### Apoptosis assay

To neutralize the apoptotic activity of agonistic anti-Fas immunoglobulin (Ig) M antibody (clone CH-11; MBL, Woburn, MA, USA), antibodies (50 ng/mL) were first incubated with recombinant human sFas (R&D Systems, Wiesbaden-Nordenstadt, Germany) for 1 hour and then added to freshly isolated neutrophils (1 × 10^6^/mL) from healthy controls. Cells were further cultured with anti-Fas antibodies in the presence of sFas for 18 hours in RPMI 1640 medium containing 2 mM glutamine (Biochrom, Berlin, Germany) and supplemented with 5% fetal calf serum (FCS) (PAA Laboratories, Coelbe, Germany), 100 U/mL penicillin and 100 μg/mL streptomycin (Invitrogen, Karlsruhe, Germany) at 37°C in a humidified atmosphere containing 5% CO_2 _before being assessed for apoptosis.

Additionally, pooled patient serum and sera immunoprecipitated with ZB4 were used to block the activity of agonistic anti-Fas antibodies (clone CH-11; 200 ng/mL). After 1 hour of incubation, patient serum (10%) containing CH-11 antibodies was added to freshly isolated control neutrophils (1 × 10^6^/mL). Cells were further cultured overnight in RPMI 1640 medium containing 2 mM glutamine (Biochrom, Berlin, Germany) and supplemented with 100 U/mL penicillin and 100 μg/mL streptomycin (Invitrogen, Karlsruhe, Germany) at 37°C in a humidified atmosphere containing 5% CO_2_.

Neutrophil apoptosis was measured by flow cytometry as the percentage of cells with fragmented DNA using the method described by Nicolleti *et al*. [26]. Briefly, cell suspensions of freshly isolated neutrophils or those incubated overnight were centrifuged at 450 × *g *for 5 minutes, and then cells were suspended in 300 μl of hypotonic fluorochrome solution (50 μg/mL propidium iodide in 0.1% sodium citrate plus 0.1% Triton X-100). Cell suspensions were stored in the dark at 4°C for at least 3 hours before they were analyzed by flow cytometry (BD Biosciences, Heidelberg, Germany). A minimum of 10,000 events were counted per sample. Results are represented as the percentage of hypodiploid DNA (sub-G1; percentage apoptosis) corresponding to fragmented DNA characteristics for apoptotic cells.

### Statistical analyses

To evaluate differences between the study groups, a Kruskal-Wallis test with Dunn's *post hoc *test was performed. Correlation between numerical values was evaluated by using Spearman's rank-correlation coefficient (*r*). Nonparametric receiver operating characteristics (ROC) curves were generated in which the value for sensitivity (true positive rate) was plotted against the false-positive rate (1 - the value of specificity). Analyses were performed using GraphPad Prism software (version 5; GraphPad Software, San Diego, CA, USA). Comparison of ROC curves was performed with MedCalc software (version 11.1.1, MedCalc Software, Mariakerke, Belgium) using the method described by Delong *et al*. [27]. Data were considered to be statistically significant at *P *< 0.05.

## Results

### Demographics and initial blood values outcomes

The 47 patients (31 male, 16 female) enrolled in this study had a mean ISS of 32.9 ± 1.7 (range, 16 to 57). The patients' mean age was 45.9 ± 2.9 years (age range, 20 to 96 years). Among all patients, 18 (38.3%) developed sepsis within 6.1 ± 0.3 days (range, 4 to 9 days) after admission. Among the septic patients, nine patients met the criteria for severe sepsis and four patients met the criteria for septic shock. The infection site of sepsis and microbiological pathogens for each patient are given in Table 1. Five patients died posttraumatically after 30.7 ± 12.3 days (range, 16 to 55 days) as a consequence of multiple organ failure (MOF). The mean ICU stay was 18.1 ± 2.6 days (range, 3 to 74 days). The mean age of the 18 patients (3 female, 15 male) who subsequently developed sepsis (sepsis group) was 53.5 ± 4.6 (range, 20 to 78 years). The mean ISS in this patient group was 36.7 ± 2.8 (range, 16 to 50). Further patient characteristics as well as injury severity and outcomes are shown in Table 2.

**Table 1:** Infection site of sepsis and microbiological pathogens

Patient	Infection site	Pathogen	Evidence for sepsis, days after trauma
1	Pneumonia	*Klebsiella pneumoniae*	4
2	Pneumonia	*Klebsiella pneumoniae*	5
3	Pneumonia	*Pseudomonas aeruginosa*	8
4	Pneumonia	*Klebsiella pneumoniae, Pseudomonas aeruginosa*	5
5	Pneumonia	*Klebsiella pneumoniae, Enterococcus faecalis*	7
6	Pneumonia	*Escherichia coli*	6
7	Pneumonia	*Morganella morganii*	6
8	Pneumonia	*Haemophilus influenzae*	4
9	Pneumonia	*Klebsiella pneumoniae*	6
10	Peritonitis	*Enterococcus faecalis*	5
11	Pneumonia	*Escherichia coli*	7
12	Pneumonia	*Pseudomonas aeruginosa*	9
13	Pneumonia	*Staphylococcus aureus*	6
14	Pneumonia	*Staphylococcus aureus*	7
15	Pneumonia	*Klebsiella pneumoniae*	7
16	Pneumonia	*Klebsiella pneumoniae*	4
17	Surgical wound infection	*Enterococcus faecalis*	5
18	Pneumonia	*Enterobacter cloacae*	8

**Table 2:** Demographics, injury severity, and outcome among subsets of patients^a^

Parameter	All patients	Nonsepsis	Sepsis
Number, *n*	47	29	18
Age, yr (±SEM)	45.9 ± 2.9	41.1 ± 4.4	53.5 ± 4.6^b^
ISS (±SEM)	32.9 ± 1.7	30.5 ± 2.0	36.7 ± 2.8^b^
ICU, days (±SEM)	18.1 ± 2.6	13.2 ± 2.9	25.9 ± 4.3^b^
Sepsis, % (*n*)	38.3 (18)	0 (0)	100 (18)
Death, % (*n*)	10.6 (5)	0 (0)	27.8 (5)
Max SOFA day 1	9.2 ± 0.6	8.4 ± 0.9	10.6 ± 0.5
Max SOFA day 5	6.2 ± 0.6	4.4 ± 0.8	9.1 ± 0.7^b^
Max SOFA day 9	4 ± 0.6	2.0 ± 0.6	7.1 ± 1.0^b^

^a^ISS, injury severity score; ICU, intensive care unit length of stay; Max SOFA, maximal Sequential Organ Failure Assessment score; ^b^*P *< 0.05 between sepsis and nonsepsis groups.

### Levels of sFas and sFasL in patients with or without sepsis after major trauma

**Figure 1:**
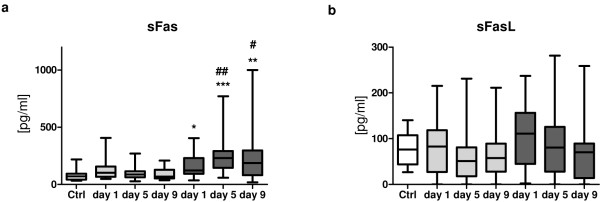
**Kinetics of serum soluble Fas (sFas) and soluble Fas ligand (sFasL) after major trauma**. **(a) **sFas levels in patients who developed sepsis during the first 10 days after trauma (*n *= 18, dark gray boxes) are significantly elevated when compared to the values determined in patients with uneventful recovery (*n *= 29, light gray boxes) and healthy volunteers (*n *= 17, white box). **(b) **No alterations in sFasL levels were observed between different groups. The horizontal line across the boxplots represents the median, and the lower and upper ends of the boxplots are the 25th and 75th percentiles, respectively. Whiskers indicate the minimum and maximum values, respectively. *P < 0.05, **P < 0.01, ***P < 0.001 vs. control group; ^#^*P *< 0.05, ^##^*P *< 0.01 vs. nonsepsis group.

Levels of sFas and sFasL were determined in the serum of healthy volunteers (control group) and patients within 24 hours after admission (day 1), at day 5 and at day 9 after major trauma (Figure 1). Patients were divided in two groups: those who subsequently developed sepsis and those with uneventful recovery after major trauma.

Within the first day after admission, sFas values of patients who subsequently developed sepsis, but not the sFas values of those with uneventful outcomes (median, 101.6; interquartile range (IQR), 66.62 to 156.9), were significantly increased (median, 122.5; IQR, 92.84 to 230.7; *P *< 0.05) compared with the healthy control group (median, 70.29; IQR, 42.9 to 93.29) (Figure 1a). Furthermore, sFas levels in these patients remarkably increased within the next days and peaked at day 5 after trauma (median, 230; IQR, 145.2 to 291.2), whereas the values for patients without development of sepsis normalized at this time point (median, 86.46; IQR, 62.95 to 114.2). sFas values in the sepsis group remained enhanced until day 9 after trauma (median, 187.1; IQR, 80.22 to 297.4) compared with values in the nonsepsis group at the same time (median, 68.95; IQR, 52.44 to 128.8). Significant intergroup differences were detectable between patients with sepsis development and healthy volunteers at day 1 (*P *< 0.05), day 5 (*P *< 0.001) and day 9 (*P *< 0.01). Additionally, sFas levels increased significantly in sepsis patients at day 5 (*P *< 0.01) and day 9 (*P *< 0.05) compared with the nonsepsis patients.

In contrast, for both groups (with or without sepsis), sFasL values were on an equivalent level compared with that of healthy controls throughout the entire observation period (*P *> 0.05) (Figure 1b) and did not show any intergroup differences.

### Prevention of neutrophil apoptosis by recombinant and serum sFas

**Figure 2:**
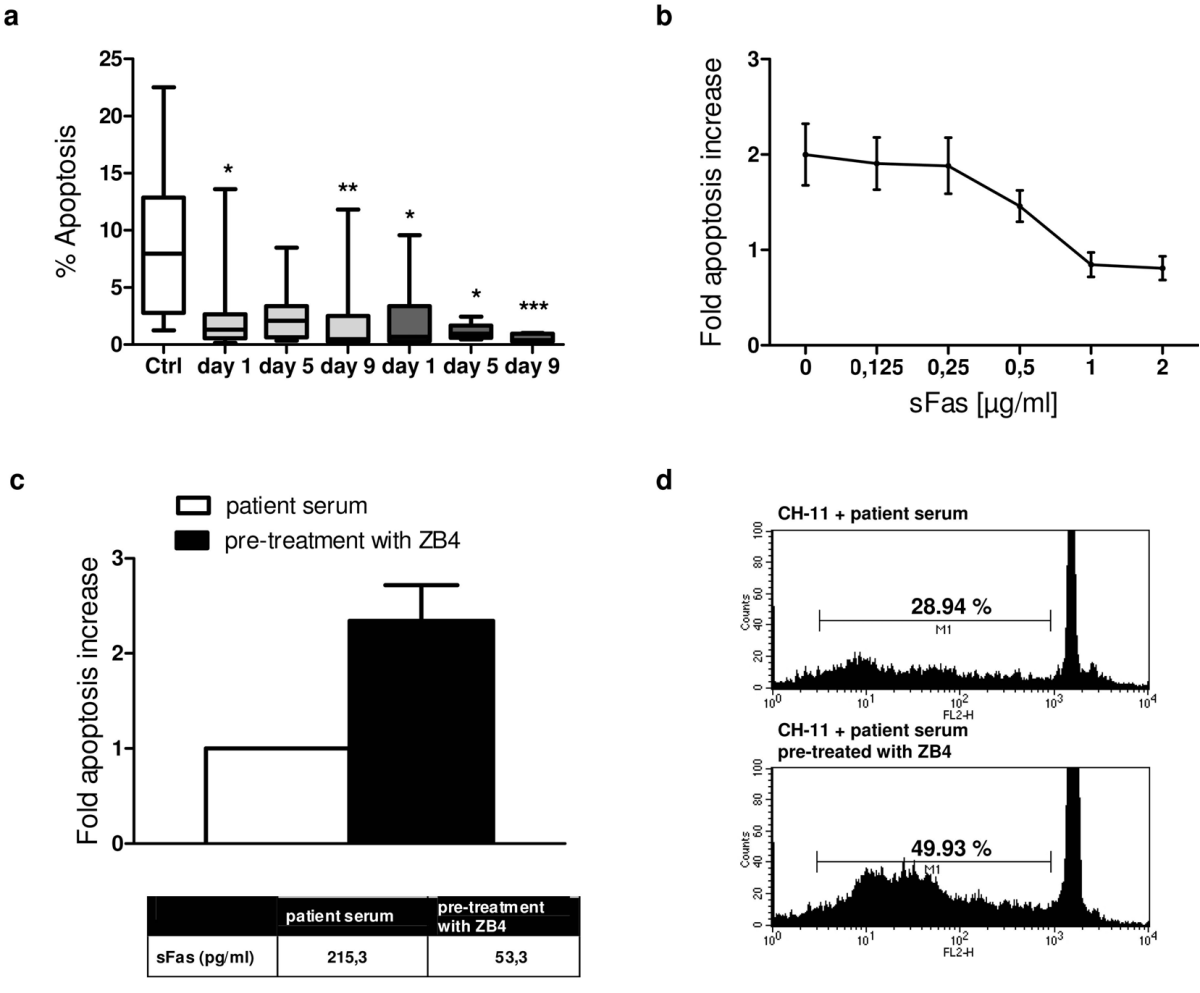
**Inhibition of neutrophil extrinsic apoptosis by sFas**. **(a) **Reduced percentage of apoptotic neutrophils isolated from healthy controls (*n *= 15, white box), sepsis patients (*n *= 7, dark gray boxes) and nonsepsis patients (*n *= 13, light gray boxes) at day 1, day 5 and day 9 after trauma. Boxplots represent the median (heavy line in boxes) and the 25th and 75th percentiles (lower and upper lines of the box, respectively). Whiskers indicate the minimum and maximum values, respectively. *P < 0.05, **P <0.01, ***P < 0.001 vs. control group. **(b) **Neutrophils from healthy controls were incubated with 50 ng/mL anti-Fas antibody (CH-11) in the presence of serial dilutions of recombinant human soluble Fas (sFas) (range, 0 to 2 μg/mL) for 18 hours. Thereafter cells were lysed in hypotonic solution containing propidium iodide, and the percentage of apoptotic cells was determined by flow cytometry. Data (means ± SEM) from three independent experiments are presented. **(c) **Control neutrophils were incubated with 200 ng/mL agonistic anti-Fas antibodies (clone CH-11) and pooled serum from four sepsis patients immunoprecipitated by anti-Fas antibodies (clone ZB4) or not. After 18 hours of culture, apoptotic neutrophils with hypodiploid DNA content were quantified by propidium iodide staining and flow cytometry. Data (means ± SEM) from six independent experiments are depicted. **(d) **Representative histogram of CH-11-induced apoptosis in the presence of patient serum. Region M1 describes the percentage of hypodiploid DNA.

It is well established that sFas may bind to membrane-bound FasL, thus blocking binding of the ligand to the Fas receptor and preventing apoptosis induction in the target cell. Therefore, we assumed that elevated serum levels of sFas may inhibit apoptosis in circulating neutrophils and promote prolonged cellular activity. Neutrophil apoptosis in both patient groups, those who developed sepsis subsequently and those with an uneventful recovery, was significantly reduced within the first day after trauma and continued to be reduced for the entire period until day 9 after trauma. The sepsis patients had a lower rate of neutrophil apoptosis at day 5 (median, 0.94; IQR, 0.6 to 1.67; vs. median, 2.08; IQR 0.64 to 3.36; in the nonsepsis group) and day 9 (median, 0.38; IQR, 0.28 to 0.94; vs. median, 0.47; IQR 0.26 to 2.5; in the nonsepsis group), although this difference did not reach the level of significance (Figure 2a).

We therefore speculated that sFas prevents the activation of Fas on trauma neutrophils, leading to strong inhibition of neutrophil extrinsic apoptosis in sepsis. To prove this hypothesis of sFas-mediated apoptosis inhibition, neutrophils from healthy donors were incubated with an agonistic anti-Fas antibody (CH-11) in the presence of serial dilutions of recombinant human sFas, which has been shown to inhibit FasL-induced apoptosis of Jurkat cells [13]. As depicted in Figure 2b, we found that sFas blocks apoptosis in a concentration-dependent manner.

We further investigated whether sFas in the sera of patients with sepsis development might also inhibit CH-11-mediated neutrophil apoptosis. Patient serum contains a broad range of cytokines, especially high levels of granulocyte macrophage colony-stimulating factor (GM-CSF), which is known to reduce the neutrophil apoptosis rate during inflammation by inhibiting the intrinsic apoptosis pathway [28]. Because serum containing high or moderate levels of sFas might also differ in the concentrations of the cytokines mentioned above, we pooled sera from four sepsis patients before immunoprecipitation of sFas by anti-Fas antibodies (ZB4). Then sera were further used to block the proapoptotic activity of CH-11 monoclonal antibodies. As depicted in Figures 2c and 2d, neutrophils incubated with agonistic CH-11 antibodies and sera from sepsis patients immunoprecipitated with ZB4 (low sFas levels) displayed a twofold increased apoptosis rate when compared with cells cultured in the presence of CH-11 antibodies and pooled serum samples (control; high sFas levels).

### Increased levels of PMNE in patients with development of posttraumatic sepsis

**Figure 3:**
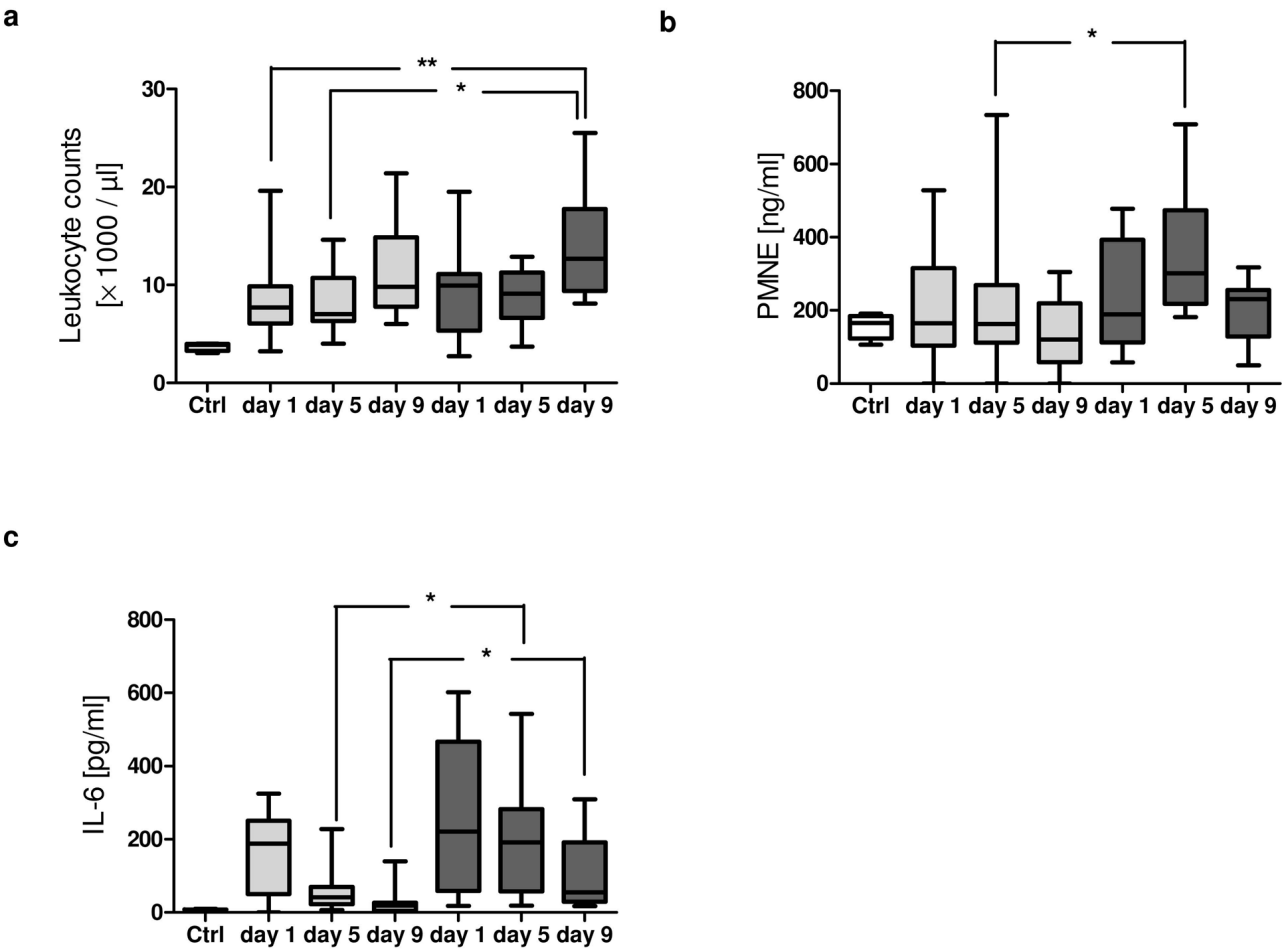
**Leukocyte count, plasma levels of neutrophil elastase (PMNE) and IL-6 levels after major trauma**. **(a) **Total leukocyte counts from controls (*n *= 4, white box), patients with sepsis development (*n *= 16, dark gray boxes) and patients with uneventful outcome (*n *= 29, light gray boxes). **(b) **PMNE in controls (*n *= 6, white box), the sepsis group (*n *= 16, dark gray boxes) and the nonsepsis group (*n *= 28, light gray boxes). **(c) **Serum IL-6 levels in trauma patients with sepsis (*n *= 18, dark gray boxes) and without sepsis (*n *= 27, light gray boxes). Boxplots represent the median (heavy line in boxes) and the 25th and 75th percentiles (lower and upper lines of the box, respectively). Whiskers indicate the minimum and maximum values, respectively. *P < 0.05, **P < 0.01.

As shown in Figure 3a, leukocyte counts were found to be significantly increased in septic patients at day 9 after trauma (median, 12.7; IQR, 9.4 to 17.75) compared with the number of leukocytes determined in the nonsepsis group at day 1 (median, 7.7; IQR 6.05 to 9.85; *P *< 0.01) and day 5 (median, 7; IQR, 6.3 to 10.7; *P *< 0.05). Neutrophil degranulation was further examined by assessing the levels of PMNE in patients' plasma (Figure 3b). PMNE showed peak levels at day 5 in patients who developed sepsis (median, 301.4; IQR, 217.5 to 474) compared with controls (median, 165.9; IQR, 123.1 to 184.4) and in patients with uneventful recovery (median, 162.8; IQR, 111.4 to 268.9; *P *< 0.05). Interestingly, PMNE values as well as leukocyte counts were found to correlate with serum sFas concentrations in the sepsis group at day 5 after trauma (*r *= 0.49; *P *< 0.05 for both).

### Relation of serum sFas levels with IL-6, SOFA and MOD scores and its prognostic value in septic patients

IL-6 is a widely accepted inflammatory parameter in response to major trauma and sepsis. Therefore, IL-6 values in patient serum were determined and correlated to the sFas values. As depicted in Figure 3c, IL-6 values of both groups were elevated at day 1 compared with control values, but decreased simultaneously on the following days. Differences were significant at day 5 and day 9 between the sepsis group (day 5: median, 191.7; IQR, 57.37 to 282.2; day 9: median, 54.94; IQR 29.51 to 191.4) and the nonsepsis group (day 5: median, 41.82; range, 22.74 to 69.43; *P *< 0.05; day 9: median, 19; IQR, 4.11 to 26.75; *P *< 0.05). In all patients, IL-6 showed a positive correlation with SOFA score at all time points as well as with the MOD score at days 5 and 9 (Table 3). Furthermore, a strong correlation was determined between IL-6 and sFas at day 5 (*r *= 0.42; *P *< 0.01) and day 9 (*r *= 0.4; *P *< 0.05), but not at day 1 after major trauma. No correlation between sFas and IL-6 values was found in patients with sepsis development (sepsis group).

**Table 3:** Correlations of sFas and IL-6 levels with the organ dysfunction scoring systems^a^

	SOFA			MODS		
		
Protein	Day 1	Day 5	Day 9	Day 1	Day 5	Day 9
sFas						
Nonsepsis	0.38^*NS*^	0.25^*NS*^	0.42^*NS*^	0.35^*NS*^	0.25^*NS*^	0.29^*NS*^
Sepsis	0.7^d^	0.62^c^	0.58^c^	0.18^*NS*^	0.56^c^	0.13^*NS*^
All	0.54^d^	0.56^d^	0.61^d^	0.28^*NS*^	0.44^c^	0.37^b^
IL-6						
Nonsepsis	0.55^b^	0.57^c^	0.21^*NS*^	0.08^*NS*^	0.15^*NS*^	0.09^*NS*^
Sepsis	0.46^*NS*^	0.33^*NS*^	0.59^c^	0.35^*NS*^	0.25^*NS*^	0.71^c^
All	0.35^b^	0.54^d^	0.57^d^	0.2^*NS*^	0.37^b^	0.43^c^

^a^SOFA, Sequential Organ Failure Assessment score; MODS, Multiple Organ Dysfunction Score; sFas, soluble Fas; IL-6, interleukin-6; *NS*, not significant; ^b^*P *< 0.05; ^c^*P *< 0.01; ^d^*P *< 0.001.

To investigate the predictive potential of sFas for the development of sepsis after major trauma, sFas values were additionally correlated to SOFA and MOD scores (Table 3). Elevated sFas concentrations determined in patients with sepsis development after severe trauma strongly correlated with patients' SOFA scores from day 1 until day 9 after trauma. In this patient cohort, sFas values at day 5 were also significantly correlated to the MOD score and were positively associated with the development of multiple organ dysfunction (Table 3). However, sFas did not correlate with SOFA and MOD scores of patients with uneventful recovery.

### ROC curves

**Figure 4:**
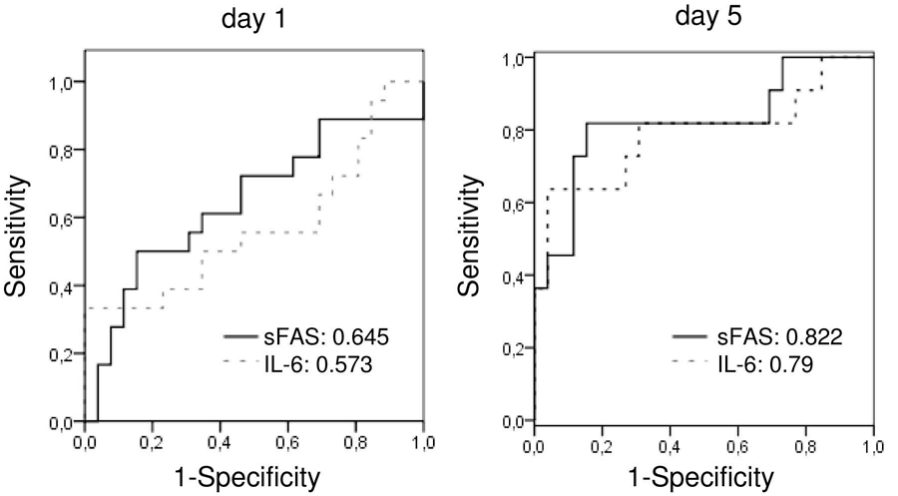
**Receiver operating characteristic (ROC) curves using sFas and IL-6 as predictors of sepsis**. The area under the curve (AUC) is given for each graph. On day 5, the seven patients who already had sepsis were excluded from the ROC curve analysis.

To verify the prognostic potential of sFas in relation to the established prognostic marker IL-6 for sepsis development after major trauma, we established ROC curves for both parameters at each time point. Figure 4 shows ROC curves of sFas and IL-6 at day 1 and day 5 after trauma. Pairwise comparison of the ROC curves displayed no statistical difference between the area under the curve (AUC) for the sFas and IL-6 values at the depicted time points (day 1, *P *= 0.694; day 5, *P *= 0.911).

## Discussion

In this study, we have demonstrated that sFas, which has been found to be significantly elevated in the sera of trauma patients who subsequently developed sepsis, inhibits the activation of the Fas pathway and thus extrinsic apoptosis induction in neutrophils.

Neutrophil apoptosis is regulated by the expression of pro- and antiapoptotic factors and might be initiated by the activation of TNF family receptors such as Fas by naturally occurring ligands such as FasL. Many proinflammatory cytokines such as GM-CSF, IL-8 and IL-6 are known to prolong neutrophil survival [29]. Recent studies have shown that proinflammatory mediators activate both the extracellular signal-regulated kinase and phosphatidylinositol 3-kinase pathways [30,31] and might trigger upregulation of antiapoptotic factors such as Mcl-1 [32], thus promoting intrinsic apoptosis resistance in neutrophils [28].

The Fas/FasL system plays a key role in maintaining the homeostasis of the immune system. It is widely accepted that sFas can protect cells against Fas-mediated apoptosis by binding to FasL, thereby functionally antagonizing the Fas-FasL pathway [13]. Evidence has been reported for a relation between elevated sFas levels and severe illness [33-37], such as sepsis [36], malignant disease [37], autoimmune diseases [13] or acute respiratory distress syndrome [38], or after major surgery [39]. It has been suggested that sFas decreases neutrophil apoptosis in patients postoperatively [39].

In the present study, the sFas levels in patients who developed sepsis were found to be significantly elevated at day 1, day 5 and day 9 after major trauma compared with levels determined in the sera of healthy donors and at day 5 and day 9 compared with patients with uneventful recovery. Our *in vitro *experiments with recombinant sFas and sera from septic patients demonstrate the abrogation of CH-11-induced neutrophil apoptosis. We have clearly shown by immunoprecipitation that the antiapoptotic effects of patient serum were largely mediated by sFas. We therefore postulate that the antiapoptotic activity of sFas in combination with the previously reported impaired intrinsic apoptosis pathway in neutrophils after trauma might be an important factor in the ongoing inflammatory injury and progressive organ dysfunction seen in sepsis patients [40,41].

Indeed, serum sFas concentrations showed a strong positive correlation with SOFA and MOD scores, especially in those patients who developed sepsis. Additionally, sFas values in patients with septic shock tended to be higher at day 5 and day 9 after trauma compared with the sFas levels in patients suffering from sepsis and severe sepsis (data not shown). Thus, our data demonstrate that sFas levels correlate with patient prognosis and might be used as an additional prognostic sepsis marker already at day 1 after trauma when sepsis is clinically not apparent. Moreover, as an interesting new aspect, we found sFas levels in patients with sepsis development to be persistently increased even at day 5 and day 9 after trauma, thus showing an association with the reduced neutrophil apoptosis found at these time points. Surprisingly, no significant differences in peripheral circulating leukocyte numbers between both patients groups could be found. This finding might be explained by the fact that activated neutrophils become rapidly recruited to the injured tissue and thus cannot be further detected in the peripheral circulation.

These data show for the first time the role of sFas as a predictor for sepsis and the potential link to neutrophil activity and the pathophysiology of major trauma. However, the ISSs of the patients in our series ranged between 16 and 57. This heterogeneity between patients in terms of injury severity as well as the small number of patients included may present potential limitations of the current study.

In contrast to the work of Papathanassoglou *et al*. [33], here sFas strongly correlated with IL-6 levels in serum from trauma patients, except for day 1. Nevertheless, no association was found between IL-6 and sFas in patients with sepsis development. IL-6 levels did not specifically correlate with SOFA and MOD scores of the sepsis group, pointing to sFas as a marker for sepsis and clinical outcome.

The highest sFas serum concentrations as well as the best correlation with leukocyte counts, PMNE, IL-6 and MODS were found at day 5 after severe trauma. Interestingly, at this time point, sepsis frequently develops clinically [8]. Because it is known that sFas may also influence the adaptive T cell-mediated immunity [42,43], it may be speculated that sFas might contribute to T cell anergy and sepsis.

In this study, reduced neutrophil apoptosis has also been observed in patients who did not develop sepsis. This finding indicates that sFas-mediated effects on neutrophils contribute to the development of organ dysfunction due to prolonged neutrophil hyperactivity, but not directly to the development of sepsis. Moreover, it is likely that sFas might additionally promote a phenotypical and functional change in neutrophils, resulting in an indirect inhibition of T cell function, which is widely accepted to be associated with sepsis development [44,45]. In this context, impairment of T cell proliferation by soluble CD83 molecules, neutrophil-derived arginase and ROS has been reported [46-48]. Nevertheless, the relationship between neutrophil hyperactivity and the extensive lymphocyte apoptosis seen in sepsis-related immunosuppression is currently incompletely understood and should be elucidated in future studies.

## Conclusions

In summary, the present study demonstrates for the first time a role of serum sFas in the inhibition of neutrophil extrinsic apoptosis associated with increased levels of PMNE, a marker for systemic inflammation. Our results show a high correlation between sFas and patients' SOFA and MOD scores in sepsis and thus provide evidence for the clinical significance of the risk for the development of sepsis and MOF. Thus, sFas may represent a feasible target for new therapeutic strategies to limit neutrophil life span and hyperactivity.

## Key messages

• Serum sFas levels have been shown to be significantly elevated in patients with sepsis development after major trauma compared with patients with uneventful recovery and healthy controls.

• Fas-mediated neutrophil apoptosis was efficiently inhibited by serum sFas from sepsis patients. Elevated sFas levels were associated with increased levels of PMNE, a marker for neutrophil activity.

• sFas showed a positive correlation with SOFA and MOD scores and sepsis development in severely injured patients.

• sFas may represent a feasible target for new therapeutic strategies to prevent neutrophil hyperactivity and sepsis.

## Abbreviations

AIS: Abbreviated Injury Scale; ARDS: acute respiratory distress syndrome; AUC: area under curve; ERK: extracellular signal-regulated kinase; FasL: Fas ligand; FCS: fetal calf serum; GM-CSF: granulocyte macrophage colony-stimulating factor; ICU: intensive care unit; IL: interleukin; IQR: interquartile range; ISS: Injury Severity Score; MOD(S): Multiple Organ Dysfunction (Score); MOF: multiple organ failure; PI3K: phosphatidylinositol 3-kinase; PBS: phosphate-buffered saline; PMNE: neutrophil elastase; ROC: receiver operating characteristics; ROS: reactive oxygen species; SEM: standard error of the mean; sFas: soluble Fas; sFasL: soluble Fas ligand; SIRS: systemic inflammatory response syndrome; SOFA: Sequential Organ Failure Assessment; TNF: tumor necrosis factor.

## Competing interests

The authors declare that they have no competing interests.

## Authors' contributions

AP-G and SF conceived the study, analysed and interpreted data and drafted the manuscript. Experimental work was performed by AP-G. TL contributed to the acquisition and analysis of patient data as well as to the writing of the manuscript. MS and JW critically revised the manuscript for intellectual content and gave important advice. All authors read and approved the final manuscript.
